# Winter survival in red clover: experimental evidence for interactions among stresses

**DOI:** 10.1186/s12870-024-05167-5

**Published:** 2024-05-28

**Authors:** Åshild Ergon, Helga Amdahl

**Affiliations:** 1 Faculty of Biosciences, Norwegian University of Life Sciences, P.O. Box 5003, Ås, N-1432 Norway; 2 Graminor AS, Hommelstadvegen 60, Ridabu, 2322 Norway

**Keywords:** Clover rot, Cold acclimation, Freezing, Growth, Resistance, *Sclerotinia trifoliorum*, Stress interactions, Tolerance, *Trifolium pratense*

## Abstract

**Background:**

There is a lack of knowledge on the combined effects of different stresses on plants, in particular different stresses that occur during winter in temperate climates. Perennial herbaceous plants in temperate regions are exposed to many different stresses during winter, but except for the fact that cold temperatures induce resistance to a number of them, very little is known about their interaction effects. Knowledge about stress interactions is needed in order to predict effects of climate change on both agricultural production and natural ecosystems, and to develop adaptation strategies, e.g., through plant breeding. Here, we conducted a series of experiments under controlled conditions to study the interactions between cold (low positive temperature), clover rot infection (caused by *Sclerotinia trifoliorum*) and freezing, in red clover (*Trifolium pratense*) accessions. We also compared our results with winter survival in field experiments and studied associations between stress and shoot growth.

**Results:**

Exposure to low positive temperatures (cold acclimation) induced resistance to clover rot. There was a clear negative interaction effect between freezing stress and clover rot infection, resulting in up to 37% lower survival rate compared to what would have been expected from the additive effect of freezing and infection alone. Freezing tolerance could continue to improve during incubation under artificial snow cover at 3 °C in spite of darkness, and we observed compensatory shoot growth following freezing after prolonged incubation. At the accession level, resistance to clover rot was negatively correlated with growth in the field during the previous year at a Norwegian location. It was also negatively correlated with the shoot regrowth of control plants after incubation. Clover rot resistance tests under controlled conditions showed limited correlation with clover rot resistance observed in the field, suggesting that they may reveal variation in more specific resistance mechanisms.

**Conclusions:**

We here demonstrate, for the first time, a strong negative interaction between freezing and infection with a winter pathogen. We also characterize the effects of cold acclimation and incubation in darkness at different temperatures on winter stress tolerance, and present data that support the notion that annual cycles of growth and stress resistance are associated at the genetic level.

**Supplementary Information:**

The online version contains supplementary material available at 10.1186/s12870-024-05167-5.

## Background

The ongoing climate change has led to a need to predict the effect of future climatic conditions on plant survival, growth and reproduction, as well as to breed agricultural crop varieties that are optimized for future conditions. These needs have made it clear that, while we have some knowledge about the effects of individual stresses, we know very little about the effects of simultaneous or sequential combinations of stresses. Plants are exposed to a range of abiotic and biotic stresses during their lifetime and have, in addition to constitutive mechanisms, evolved responses that enable them to cope with these stresses. Signalling pathways eliciting such responses interact in an extensive signalling network that integrates external (environmental) and internal (developmental) stimuli and governs the allocation of resources to protective responses, storage, growth and development. Interacting effects of stresses on plants can occur at many different levels from gene to physiology and morphology. These effects may also vary depending on other environmental factors, plant developmental stage, individual stress levels and durations, and on whether the stresses occur simultaneously or sequentially. The effects of stress combinations, and particularly the activation of signalling pathways, have attracted some attention in the past decade and is the subject of several review papers [1–3].

Overwintering herbaceous plants face a number of different abiotic stresses during the winter, along with psychrophilic pathogens [4]. A snow cover established before the ground is deeply frozen will promote fungal disease but protects plants against freezing, which will be more harmful if temperatures drop without a snow cover in place. During the winter a snow cover may come and go due to variations in weather conditions and thus plants may be exposed to different stresses at different times during the winter period.

Winter survival is a major limitation for plant species at high latitudes, including agricultural biennial or perennial crops. Overwintering species adapted to cold climates have evolved a cold acclimation response, whereby they go through a developmental and physiological reprogramming in response to lower temperatures and shorter photoperiods in the autumn [5–7]. The cold acclimation is necessary for these plants to survive winter. Not only freezing tolerance is substantially improved by cold acclimation, but also resistance to other winter stresses, such as winter pathogens [8–11].

Due to a number of complicating factors, there are large uncertainties regarding the effects of climate change on winter stresses and plant survival and climate change can both worsen and alleviate winter stresses [12–14]. For example, higher winter temperatures may lead to a reduction in the presence of a protective snow cover, resulting in more freezing stress, and more precipitation in winter and unstable weather conditions is expected to result in more ice cover, anoxia and more frequent freeze-thaw events. However, in cold areas where winter temperatures will still remain below 0 °C, more precipitation may lead to deeper and longer-lasting snow cover. Indirect effects can also occur, for example, a shift of the cold acclimation period to a time of the year with less light may have a negative effect on the cold acclimation process, depending on the importance of light for the cold acclimation process of the species in question [13].

Improved winter survival is a major breeding goal in most red clover breeding programs [15, 16]. Clover rot, caused by *Sclerotinia trifoliorum* infection of the root and crown during winter, is regarded as an important factor leading to winter mortality [17, 18]. There is, however, strong genotype x environment interactions on red clover winter survival, and in some locations and years freezing tolerance plays a role [19]. Genetic variation in winter survival, freezing tolerance and clover rot resistance has been described [20–24]. Red clover has been shown to be more resistant to clover rot after a period of cold acclimation than before, both when incubated under humid conditions in a greenhouse [22, 25] or under an artificial snow cover in darkness at a low positive temperature [17]. To our knowledge the effect of the length of the cold acclimation period on resistance to clover rot has so far not been investigated. Apart from the knowledge that a mild cold stress induces some resistance to several different winter stresses, there is generally very limited information about interactions among different winter stresses on plants. In theory, such interactions could have both positive and negative effects on winter survival, as one type of stress may either elicit responses that protect against other types of stress, or cause damage that renders plants more vulnerable to other types of stresses. Besides being informative for development of plant breeding strategies, knowledge on winter stress interactions and the physiology behind could help improving prediction models of grassland growth and overwintering [26].

This research includes experiments aimed at answering the following two main questions: (i) does a longer period of cold acclimation increase resistance to clover rot? and (ii) is there an interaction between clover rot and freezing stress? For this, the responses of several European red clover accessions were tested. In addition, by including results from previous studies similar red clover material we investigated associations between resistance to clover rot and shoot growth potential.

## Results

Four experiments were performed under controlled conditions. In experiment 1 and 2, we investigated the effects of various factors on resistance to clover rot, namely cold acclimation (both experiments), length of cold acclimation (both experiments) and plant age (experiment 1) prior to freezing and/or inoculation with clover rot, as well as length of incubation and incubation temperature under artificial snow cover following clover rot inoculation (experiment 2). In experiment 3 and 4 we studied the interactions between freezing stress and clover rot. All experiments except experiment 2 included non-inoculated, but incubated controls in order to also study the effect of incubation stress per se. Experiment 3 and 4 included treatments with freezing stress applied either before or after inoculation and/or incubation, in order to study if the order of the stress treatments mattered. See Table 1, Supplementary Table 1 and Supplementary Fig. 1 for a comparative overview of the growth, inoculation, incubation and freezing treatments in the different experiments, and the Methods section for details.

**Table 1:** Overview of experiments. Further details on the organization of experiments are given in Supplementary Table 1

	Experiment 1	Experiment 2	Experiment 3	Experiment 4
Research questions	• Does cold acclimation (CA) induce resistance to clover rot? If so, does the length of CA matter? • Do accessions differ in resistance to clover rot? Do they differ in any CA-dependent resistance?	• Does cold acclimation (CA) induce resistance to clover rot? If so, does the length of CA matter? And is CA-induced resistance expressed in our experiments simply due to the lack of a cold-shock upon incubation? • Do accessions differ in resistance to clover rot? Do they differ in any CA-dependent resistance? • How well does resistance expressed at 16 °C correlate with resistance expressed at 3 °C?	• Is there an interaction (synergistic or antagonistic) between clover rot and freezing stress?	
Growth treatments	NA-YOUNG: 6 w^1^ NA^2^ NA-OLD: 9 w NA CA-SHORT: 6 w NA + 1 w CA^3^ CA-LONG: 6 w NA + 3 w CA	NA: 7 w NA CA-SHORT: 6 w NA + 1 w CA CA-LONG: 6 w NA + 3 w CA	6 w NA + 3 w CA	6w NA + 3w CA
	Greenhouse during winter, set at 16 °C, 12 h photoperiod, natural light supplied with 150 µmol m^−2 ^s^−1^	Growth chamber, 16 °C, 12 h photoperiod, 100 µmol m^−2 ^s^−1^	Greenhouse during winter, set at 16 °C, 12 h photoperiod, natural light supplied with 150 µmol m^−2 ^s^−1^	Growth chamber, 16 °C, 12 h photoperiod, 100 µmol m^−2 ^s^−1^
	Growth chamber, 3 °C, 12 h photoperiod, 100 µmol m^−2 ^s^−1^	Growth chamber, 3 °C, 12 h photoperiod, 100 µmol m^−2 ^s^−1^	Growth chamber, 3 °C, 12 h photoperiod, 100 µmol m^−2 ^s^−1^	Growth chamber, 3 °C, 12 h photoperiod, 100 µmol m^−2 ^s^−1^
Inoculation treatments	Non-inoculated, inoculated	Inoculated	Non-inoculated, inoculated	Non-inoculated, inoculated
Incubation under artificial snow cover	3 °C, 5.5 w 3 °C, 7.5 w	16 °C, 1 w 16 °C, 2 w 16 °C, 3 w 3 °C, 6 w 3 °C, 7 w 3 °C, 8 w	3 °C, 3 w 3 °C, 6 w	3 °C, 3 w 3 °C, 4.5 w
Freezing treatments before or after inoculation and incubation	None	None	No freezing -4.5 °C before -6 °C before -4.5 °C after -6 °C after	No freezing -4.5 °C before -6 °C before -7.5 °C before -4.5 °C after -6 °C after -7.5 °C after
Analyzed response variables	Survival, DW^4^ of regrowth, relative DW of regrowth	Survival	Survival, DW of regrowth	Survival, DW of regrowth

^1^weeks; ^2^no cold acclimation; ^3^cold acclimation; ^4^dry weight

### Experiment 1 and 2: variation in resistance to clover rot

**Fig. 1:**
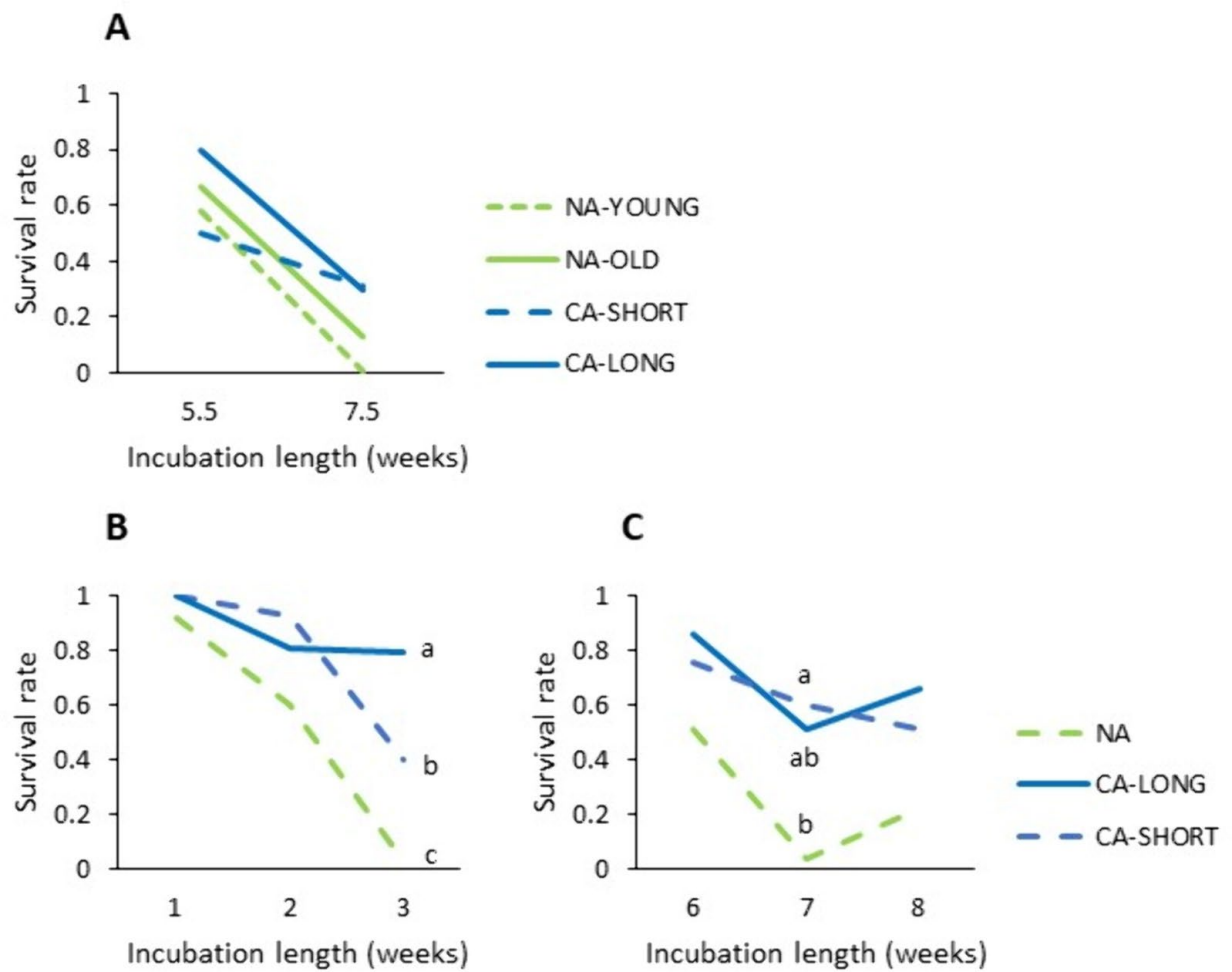
Survival rate of red clover from different growth treatments after inoculation with *Sclerotinia trifoliorum* and incubation under artificial snow cover at 3 °C in experiment 1 (**A**) and at 16 °C (**B**) or 3 °C (**C**) in experiment 2. Averages of 9 (A) and 12 (B, C) populations are shown (see Table 2 for an overview). NA-YOUNG, NA and NA-OLD; non-acclimated plants that were 6, 7 or 9 weeks old, respectively. CA-SHORT and CA-LONG; 6 weeks old plants cold acclimated for one or three additional weeks, respectively. Least Square Means generated in analyses of variance (Supplementary Tables 3 and 3) are shown. Values within panels and time points that are not labelled with the same letter are significantly different (*P* < 0.05)

A period with low positive temperatures (cold acclimation) had a strong positive effect on survival of inoculated plants in experiment 2 (Fig. 1B and C) and a weaker effect in experiment 1 (Fig. 1A). There was no clear effect of plant age, and a longer cold acclimation treatment only improved survival at the high incubation temperature (16 °C), and not at the low incubation temperature (3 °C).

**Table 2:** Resistance to clover rot measured in experiment 1 (A, incubation under artificial snow cover at 3 °C) and 2 (B, incubation under artificial snow cover at 16 °C). LS means obtained from analyses of variance (Supplementary Table 2 A, 2D, 3A) are presented

A			B	
Population	Survival rate^1^	Relative regrowth^1^	Population	Survival rate^1^
NGB2487	0.65^a^	0.67^a^	SWÅ RK09093 (Åke)	0.79^a^
Saija	0.44^ab^	0.51^ab^	LøRk9627	0.77^ab^
Gandalf	0.45^ab^	0.50^ab^	GnRk0747	0.76^ab^
S592 AberChianti	0.44^ab^	0.47^ab^	SW RK1120	0.75^ab^
Vltavín	0.38^b^	0.45^ab^	Discovery	0.74^ab^
Sangria	0.39^b^	0.44^ab^	GnRk0729	0.72^ab^
Karim	0.33^b^	0.38^ab^	TPD-05-16-3177	0.72^ab^
NGB4089	0.38^b^	0.36^ab^	TPD-05-15-3127	0.69^ab^
Trubadur	0.36^b^	0.33^b^	Lanzenhaeusern_291	0.65^ab^
			SW RK1119	0.64^ab^
			Callisto	0.63^ab^
			Niederwangen_262	0.62^b^

^1^Populations not followed by the same letter are significantly different (*P* < 0.05)

Some differences in survival of inoculated plants among accessions could be detected, but only across, and not within, the applied growth treatments. NGB2487 had a significantly higher survival rate than Sangria, NGB4089, Vltavín, Trubadur and Karim in experiment 1 (Table 2A). NGB2487 also had a significantly higher relative regrowth (inoculated vs. non-inoculated) than Trubadur. In experiment 2, a significant effect of population was seen at 16 °C only, with SWÅ RK09093 (Åke) having better survival than Niederwangen_262 (Table 3B). Survival rate at 3 °C was only moderately correlated with survival rate at 16 °C (*R* = 0.53, *P* < 0.0001).

**Fig. 2:**
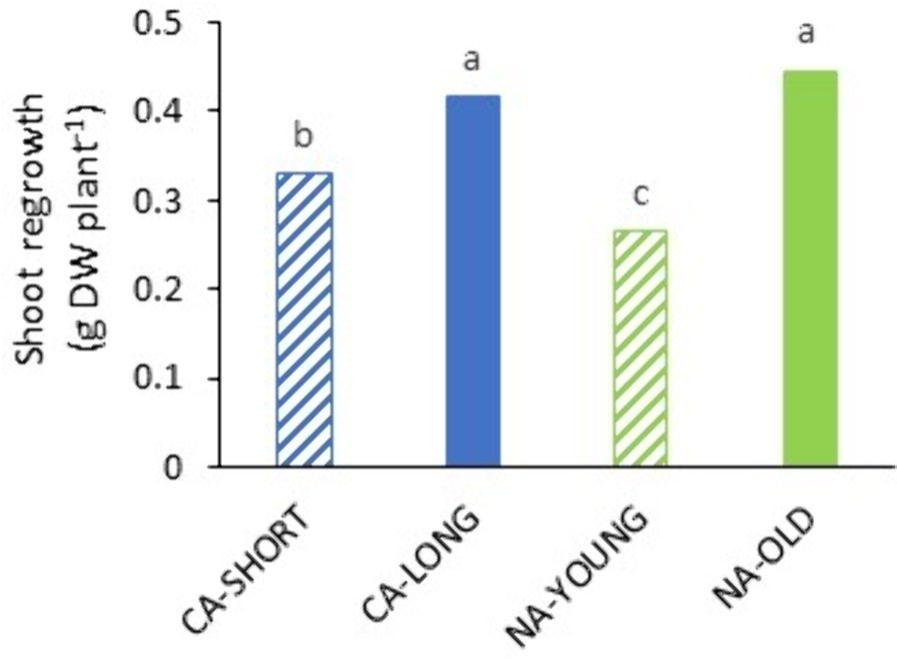
Differences in regrowth among non-inoculated plants from different growth treatments after incubation under artificial snow cover at 3 °C in experiment 1. NA-YOUNG; non-acclimated 6 weeks old plants, NA-OLD; non-acclimated 9 weeks old plants, CA-SHORT; 6 weeks old plants cold acclimated for an additional week, CA-LONG, 6 weeks old plants cold acclimated for an additional 3 weeks. LS means across populations and incubation length generated in analyses of variance (Supplementary Table 2B) are shown. Values that are not labelled with the same letter are significantly different (*P* < 0.05)

We also noted a strong effect of plant age and/or cold acclimation on regrowth of non-inoculated plants after incubation under artificial snow cover; on average across populations, 9 weeks old plants (either cold acclimated or not) had better regrowth than 6–7 weeks old plants (Fig. 2).

### Experiment 3 and 4: interaction effects between clover rot and freezing stress

When applied as single stresses, clover rot and freezing had small but significant effects on survival rates in experiment 3. Clover rot reduced survival by 11% and freezing at the lowest temperature (-6 °C) prior to incubation reduced survival by 14% (Table 3A). The two stresses had a stronger effect on the dry matter of the regrowth than on survival; when applied as single stresses they reduced the average dry weight per plant by 21% and 17–43% after 6 weeks incubation, respectively (Table 3B). In experiment 4, plants were on average more stress susceptible than in experiment 3 and there were significant effects of clover rot and freezing treatment on survival after both 3 and 4.5 weeks long incubation. In most cases, survival and dry weight of the regrowth was lower after 4.5 weeks incubation in experiment 4 than after 6 weeks incubation in experiment 3 (Table 3).

**Table 3:** Average survival rate (A) and dry weight of the regrowth (g plant^−v^) (B) following the various stress treatments and lengths of incubation under artificial snow cover in experiment 3 and 4. Percent reduction relative to the non-inoculated, non-frozen controls are given in parentheses

		Experiment 3				Experiment 4			
Inoculation treatment	Freezing treatment	3 weeks incubation		6 weeks incubation		3 weeks incubation		4.5 weeks incubation	
**A**									
Non-inoculated	None	1^a1^	-	1^a^	-	1^A^	-	0.99^A^	-
	-4.5 °C before incubation	1^a^	(0%)	1^a^	(0%)	0.96^AB^	(-4%)	0.95^AB^	(-5%)
	-6 °C before incubation	0.92^ab^	(-8%)	0.86^ab^	(-14%)	0.81^ABC^	(-19%)	0.85^AB^	(-14%)
	-7.5 °C before incubation	-		-		0.73^ABC^	(-27%)	0.47^CD^	(-53%)
	-4.5 °C after incubation	1^a^	(0%)	1^a^	(0%)	0.94^AB^	(-6%)	0.99^A^	(0%)
	-6 °C after incubation	1^a^	(0%)	1^a^	(0%)	0.96^AB^	(-4%)	0.98^A^	(-1%)
	-7.5 °C after incubation	-		-		0.81^ABC^	(-19%)	0.98^A^	(-1%)
Inoculated	None	1^a^	(0%)	0.89^a^	(-11%)	0.89^AB^	(-11%)	0.69^ABC^	(-30%)
	-4.5 °C before inoculation and incubation	0.97^ab^	(-3%)	0.53^c^	(-47%)	0.48^CD^	(-52%)	0.24^DEF^	(-76%)
	-6 °C before inoculation and incubation	0.97^ab^	(-3%)	0.44^c^	(-56%)	0.44^CDE^	(-56%)	0.13^DEF^	(-87%)
	-7.5 °C before inoculation and incubation	-		-		0.02^F^	(-98%)	0.04^EF^	(-96%)
	-4.5 °C after inoculation and incubation	0.97^ab^	(-3%)	0.46^c^	(-54%)	0.81^ABC^	(-19%)	0.77^ABC^	(-22%)
	-6 °C after inoculation and incubation	0.86^b^	(-14%)	0.61^bc^	(-39%)	0.69^ABC^	(-31%)	0.59^BC^	(-40%)
	-7.5 °C after inoculation and incubation	-		-		0.69^ABC^	(-31%)	0.67^ABC^	(-33%)
Significant effects^2^	Inoculation	NS		***		***			
	Freezing	NS		**		***			
	Incubation x Freezing	-		-		*			
	Inoculation x Freezing	*		*		***			
	Incubation x Inoculation x Freezing	-		-		**			
**B**									
Non-inoculated	None	0.53^a1^	-	0.69^a^	-	0.65^A^	-	0.39^BC^	-
	-4.5 °C before incubation	0.49^a^	(-7%)	0.58^ab^	(-17%)	0.26^CDEF^	(-60%)	0.13^EFGH^	(-67%)
	-6 °C before incubation	0.30^bc^	(-44%)	0.39^bcd^	(-43%)	0.12^EFGH^	(-81%)	0.12^FGH^	(-70%)
	-7.5 °C before incubation	-		-		0.08^GH^	(-88%)	0.07^GH^	(-83%)
	-4.5 °C after incubation	0.49^a^	(-8%)	0.47^abc^	(-32%)	0.23^DEF^	(-65%)	0.40^BC^	(0%)
	-6 °C after incubation	0.43^ab^	(-19%)	0.45^bc^	(-35%)	0.22^DEFG^	(-66%)	0.35^CD^	(-12%)
	-7.5 °C after incubation	-		-		0.13^EFGH^	(-80%)	0.40^BC^	(+ 2%)
Inoculated	None	0.52^a^	(-2%)	0.55^ab^	(-21%)	0.50^AB^	(-23%)	0.25^DEF^	(-37%)
	-4.5 °C before inoculation and incubation	0.47^a^	(-12%)	0.29^cde^	(-57%)	0.09^FGH^	(-86%)	0.03^H^	(-93%)
	-6 °C before inoculation and incubation	0.26^c^	(-51%)	0.14^e^	(-79%)	0.05^GH^	(-92%)	0.01^H^	(-97%)
	-7.5 °C before inoculation and incubation	-		-		0.00^H^	(-100%)	0.00^H^	(-99%)
	-4.5 °C after inoculation and incubation	0.41^ab^	(-23%)	0.19^de^	(-72%)	0.22^DEFG^	(-66%)	0.26^CDE^	(-33%)
	-6 °C after inoculation and incubation	0.32^bc^	(-40%)	0.25^cde^	(-64%)	0.10^FGH^	(-85%)	0.35^EFG^	(-12%)
	-7.5 °C after inoculation and incubation	-		-		0.11^FGH^	(-84%)	0.26^CDE^	(-34%)
Significant effects^2^	Inoculation	NS		***		**			
	Freezing	***		***		***			
	Incubation x Freezing	-		-		***			
	Incubation x Population	-		-		**			
	Inoculation x Freezing	NS		NS		*			
	Incubation x Inoculation x Freezing	-		-		*			

^1^Values within (experiment 3) or across (experiment 4) incubation lengths not followed by the same letter are statistically significant (*P* < 0.05)

^2^Data from experiment 3 were analysed within incubation length due to different environmental conditions in the greenhouse during regrowth; factors and interactions with no significant effects are not shown, see Supplementary Tables 5 and 6 for details of the analyses; *NS *not significant; ***, *P* < 0.0005; **, *P* < 0.005; *, *P* < 0.05

The combination of clover rot and freezing before inoculation reduced survival significantly more than what would have been expected from additive effects of the two stresses applied separately, both in experiment 3 and 4 (Table 3A, 4). In experiment 3, this was also the case for the combination of clover rot and freezing *after* inoculation, while it was not in experiment 4. In experiment 3, pre- and re-growth occurred in a greenhouse under somewhat different conditions. In particular, the natural light must have given higher light intensities than in the growth chambers used in experiment 4, where in fact long petioles were noted after pre-growth, and this may have influenced the results (see Discussion). Combining stresses had a very different effect on the dry weight of the regrowth after incubation than it had on survival. In contrast to the effect on survival, the plants exposed to both clover rot and freezing stress had a slightly higher regrowth than what would have been expected from additive effects of the two stresses applied separately (Table 3B, 4).

**Table 4:** Pairwise t-test of the difference between the observed reduction in combined stress treatments relative to the control and the expected reduction due to additive effects of the two stresses (i.e., the non-additive effect of combining stresses). The difference between each stress treatment and the non-infected and non-frozen controls were first calculated. The reduction in combined treatments were then compared with the expected additive reduction, calculated as the sum of the reduction in treatments where the two stresses were applied separately. Values were averaged across replicate snow covers, resulting in 16 and 12 pairs to compare in each test in experiment 3 and 4, respectively. When the difference indicated is larger than zero then the combined effect is larger than the additive effect and vice versa

Response variable	Time of freezing	Experiment	Non-additive effect	d.f.	t-value	*P*-value
Survival rate	Before	3	0.16	15	2.52	0.02
	Before	4	0.37	11	7.77	< 0.0001
	After	3	0.22	15	3.38	0.004
	After	4	0.03	11	1.14	0.3
DW of regrowth (g plant^−1^)	Before	3	-0.050	15	-0.71	0.4
	Before	4	-0.046	11	-2.89	0.01
	After	3	-0.017	15	-0.24	0.7
	After	4	-0.051	11	-2.62	0.02

**Fig. 3:**
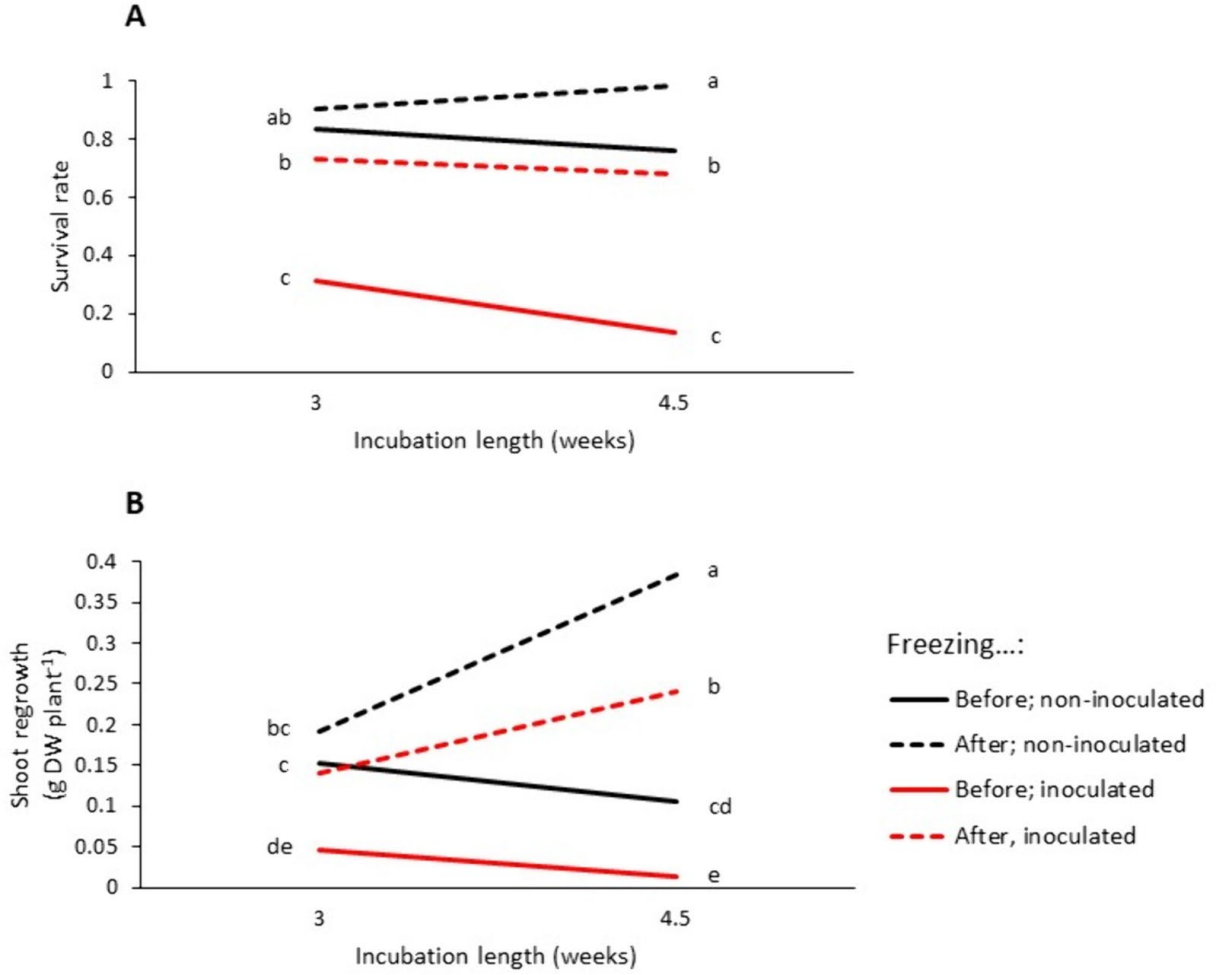
Interactions between freezing time (before or after incubation under artificial snow cover at low temperature, and averaged across freezing temperatures), inoculation with *Sclerotinia trifoliorum* and incubation length on red clover survival (**A**) and regrowth (**B**) after incubation in experiment 4. Values within panels that are not labelled with the same letter are significantly different (*P* < 0.05). See Supplementary Table 7 for the statistical analysis

In experiment 4, freezing before incubation had a stronger negative effect on survival and regrowth than freezing after incubation, both in non-inoculated and inoculated plants (Table 3; Fig. 3). After the 4.5 weeks incubation period, freezing of non-inoculated plants after incubation, even down to -7.5 °C, did not affect survival at all (Table 3A), suggesting an induction of freezing tolerance during incubation. In fact, regrowth of plants given a freezing treatment after incubation was higher in the 4.5 weeks incubation treatment (0.35–0.40 g DW plant^−1^) than in the 3 weeks incubation treatment (0.13–0.23 g DW plant^−1^) (Table 3B, Fig. 3B), even though the regrowth capacity of the control plants was reduced from 0.65 g DW plant^−1^ to 0.39 g DW plant^−1^ over the same incubation period (Table 3B), suggesting a stimulation of shoot growth induced by freezing.

### Comparison with results from other studies of the EUCLEG red clover collection

In the set of 110 EUCLEG accessions, clover rot resistance measured in the field was correlated with the clover rot resistance of non-cold acclimated plants under controlled conditions reported by Frey et al. [24], but to a limited extent (*R* = 0.34, *P* = 0.0003, Supplementary Table 8 A). In the smaller sets of accessions tested in experiment 1 and 2 there were higher correlation coefficients between survival rate of inoculated plants (across all treatments) and the clover rot resistance reported by Frey et al. [24] (*R* = 0.61–0.76, *P* < 0.04), but no correlation with clover rot resistance in the field (Supplementary Table 8B and C).

**Fig. 4:**
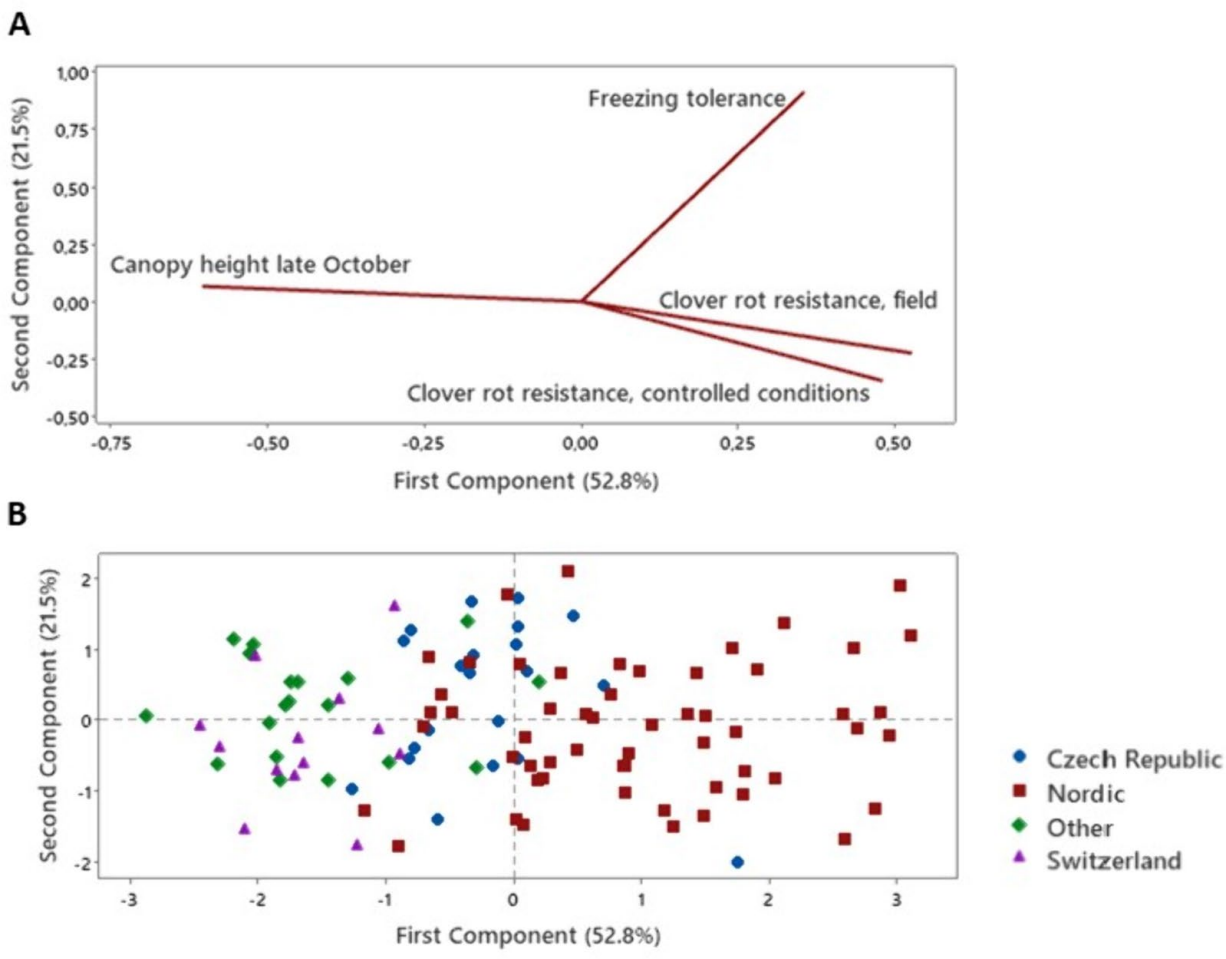
Principal component analysis of traits recorded in 110 accessions from the EUCLEG red clover panel, grouped into four regions of origin. Canopy height in late October and clover rot resistance expressed during the following winter was recorded in the Norwegian EUCLEG field experiment [23, 27], clover rot resistance data are from Frey et al. [24] and freezing tolerance data from Zanotto et al. [28]. Correlation coefficients between traits are given in Supplementary Table 8A

**Fig. 5:**
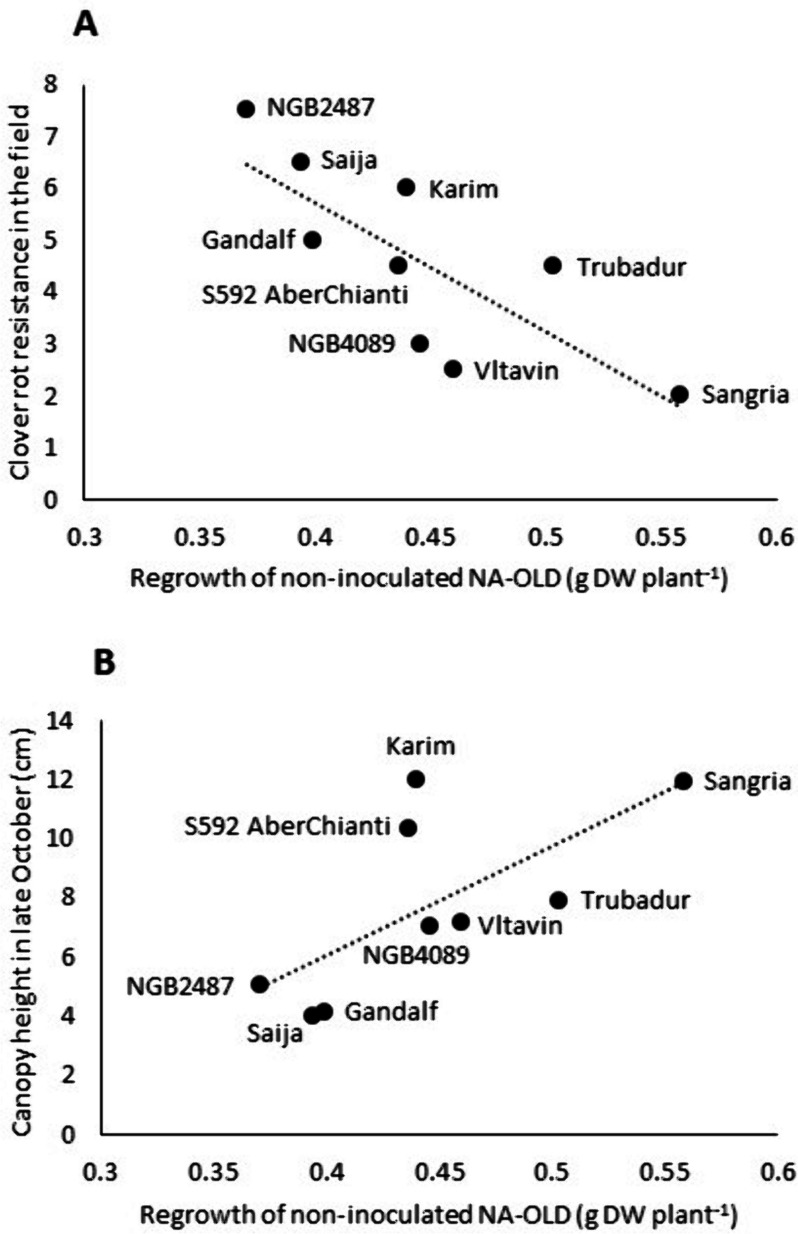
Correlation between regrowth of non-acclimated and non-inoculated (but incubated) plants in experiment 1 with canopy height in late October and clover rot resistance expressed during the following winter in the Norwegian EUCLEG field experiment [23, 27]. Correlation coefficients between traits are given in Supplementary Table 8B

Interestingly, shoot growth during the establishment year in the field experiment was negatively associated with clover rot resistance, particularly in the field experiment itself (|R|= 0.47–0.59, *P* < 0.0001, Supplementary Table 8 A), but also in the test under controlled conditions by Frey et al. [24] (|R|= 0.37–0.50, *P* < 0.0001, Fig. 4A). Shoot growth was also to a limited extent negatively correlated with freezing tolerance (|R|= 0.29–0.34, *P* < 0.002). Correlations were significant and of similar magnitude not only for canopy height in both late September and late October, but also for the difference in height between these two time points, showing that at least some of the variation in growth is due to late autumn growth. This variation was associated with geographic origin of the accessions, with Nordic material being more resistant to clover rot and having less shoot growth in the establishment year (Fig. 4B). Regrowth of non-inoculated NA-OLD plants measured in experiment 1 was also positively correlated with shoot growth in the establishment year and negatively correlated with clover rot resistance in the field experiment (|R|=0.68–0.77, *P* < 0.04; Supplementary Table 8B, Fig. 5).

## Discussion

### The effect of cold acclimation on resistance to clover rot

Our results indicate that cold acclimation before infection increases resistance to clover rot during prolonged incubation with the fungus (Fig. 1), as previously observed [17, 22, 25]. The results also show that the higher resistance in cold acclimated plants is not simply due to avoidance of a “cold shock” upon incubation at low temperature, since the effect of cold acclimation was very clear under incubation at 16 °C. The length of cold acclimation mattered only when the inoculated plants were incubated at 16 °C rather than at 3 °C, possibly due to a higher amount of carbohydrate reserves that would be needed for respiration under the dark and warm conditions, and accumulation of such compounds during cold acclimation [29]. Thus, the results indicate that the cold acclimation-induced resistance to clover rot expressed at around 3 °C (similar to natural conditions), is at least partly relying on relatively rapid cold-induced responses, such as the expression of pathogenesis-related proteins observed in many species [10, 30, 31], and less on slower responses that builds up over several weeks, such as accumulation of organic reserves in the crown tissue. It should be noted, however, that the result might have been different with a higher light intensity during CA.

### Clover rot resistance is associated with lower shoot growth potential

The negative correlation between growth in the establishment year and clover rot resistance both in the field experiment and under controlled conditions (Fig. 4A), the correlation between clover rot resistance in the field and shoot regrowth after incubation in experiment 1 (Fig. 5), as well as the association of these traits with the latitudinal origin of the accessions (Fig. 4B), suggest that adaptation to seasonal climatic variation and appropriate regulation of growth and allocation is important for clover rot resistance under field conditions at Nordic latitudes, although the traits may simply be co-inherited and not necessarily functionally related. Similarly, negative phenotypic correlations between growth in the establishment year and winter survival in two of three locations, as well as with freezing tolerance, were found in Nordic red clover gene bank material [23]. This is probably a reflection of the growth-stress tolerance trade-off which is observed in many perennial forage species [13, 32–34], but that may be at least partly genetically uncoupled from more specific stress resistance mechanisms, as shown for lucerne [35–37] and cocksfoot [32]. We found relatively low correlations or no significant correlation at all between resistance measured in the field experiment and resistance measured in several experiments under controlled conditions. It is likely that the latter reveals variation in more specific resistance mechanisms, while the former reveals the variation that is associated with shoot growth potential, in addition to variation in other winter stresses or other factors like competition, soil conditions etc. Therefore, screening of resistance under controlled conditions can supplement screening in field trials.

### Low positive temperatures can induce freezing tolerance in the absence of light

In experiment 4, freezing before incubation under artificial snow cover had a much bigger negative effect on both survival and regrowth than freezing after incubation, independently of whether plants had been inoculated with clover rot prior to incubation or not (Table 3). This was very clear after the 4.5 weeks long incubation but there was also a tendency after 3 weeks. At the same time, there was no synergistic effect between clover rot infection and freezing *after* incubation in this experiment. In fact, plants tolerated freezing much better after incubation in darkness at a low positive temperature (almost no mortality among non-inoculated plants, even at the lowest freezing temperature), suggesting that the cold acclimation process continued during the low temperature incubation and improved freezing tolerance further. Even of cold acclimation to some extent depends on the presence of light [13, 38], our results indicate that light-independent cold acclimation can also increase freezing tolerance in red clover. Whether this has any role under natural conditions is yet to be shown. With climate change it is increasingly relevant at high latitudes where the cold acclimation period is shifting towards a much darker part of the year [13]. It is puzzling that we did not observe the same phenomenon in experiment 3. The likely higher light intensities in the greenhouse used in experiment 3 compared to the growth chambers used in experiment 4 may have provided plants in experiment 3 with more organic reserves after pre-growth than in experiment 4, which may have affected stress tolerance positively and sufficiently to mask a positive effect of incubation on freezing tolerance. This explanation is supported by the fact that inoculation had a much more detrimental effect on survival in experiment 4 than in experiment 3, especially when taking the shorter incubation length into account (Table 3A).

### Freezing stress increases susceptibility to subsequent clover rot infection in a synergistic manner

We observed a clearly negative interaction between freezing stress and subsequent clover rot infection on survival (Tables 3 and 4), suggesting that under field conditions, the presence of both will exacerbate winter mortality. Being a necrotrophic pathogen, *S. trifoliorum* likely benefits from the cell and tissue damage that freezing can generate, as this will make nutrients and entries for infection available for the fungus.

### Freezing can induce compensatory shoot growth

The strong negative synergistic effect of clover rot and freezing on survival rate was not seen in the regrowth data (Table 4). There was, in fact, an opposite effect seen in experiment 4. A partial explanation may be that there is more growth per surviving plant due to less competition for light in treatments with high mortality. This could compensate for the synergistic effect between freezing and infection, but not the significant overcompensation that was found in experiment 4 (Fig. 3). Such overcompensation can be explained by a stress-induced stimulation of subsequent shoot growth. This is what we observed in plants that were frozen after incubation in experiment 4; they had more regrowth than the control plants. Compensatory growth following freezing stress has also been observed in timothy [39].

## Conclusions

We have demonstrated several interaction effects among different winter stress factors in red clover: (i) A low positive temperature prior to infection improves resistance to clover rot while (ii) freezing prior to infection results in increased susceptibility in a synergistic manner. (iii) Freezing tolerance can improve over several weeks in darkness at low positive temperatures. Moreover, we have identified associations between stresses and shoot growth, supporting the notion that annual cycles of growth and stress resistance are linked: (i) During prolonged incubation in darkness at low positive temperatures, and/or in response to freezing, subsequent compensatory shoot growth (when exposed to normal growing conditions) can be stimulated. (ii) In a diverse collection of accessions, clover rot resistance measured in the field is associated with less shoot growth prior to winter (under Nordic conditions) and immediately after a simulated winter. Finally, we found that measurements of resistance under controlled conditions were only moderately correlated with resistance measured in the field, and may therefore, in line with the above results, have revealed variation in other and more specific resistance mechanisms that are independent of annual growth cycles. Our results improve the current knowledge on winter physiology of red clover and provide information that can be used in modelling of climate effects on winter survival and productivity, as well as breeding for winter survival and climate adaptation in red clover.

## Methods

### Experiments under controlled conditions

#### Plant material, pre-growth and cold acclimation

The populations used in the experiments (Supplementary Table 2) were all diploid and belonged to the set of populations characterized in the EUCLEG project (www.eucleg.eu) [27]. The populations were selected to represent a broad range from susceptible to resistant to clover rot according to observations in the Norwegian EUCLEG field trial. Seeds were scarified with sandpaper and sown in a peat soil. After germination, individual young seedlings were transplanted into a small volume (28 cm^3^) of peat soil. In experiment 1 and 3, plants were grown in a greenhouse (59°40’ N, 10°47’ E, from November 2019) at 16 °C with natural light supplied with metal halide lamps (approximately 150 µmol m^−2^s^−1^ PAR) for a photoperiod of 12 h. Plants in experiment 2 and 4 were pre-grown in a growth chamber with approximately 100 µmol m^−2 ^s^−1^ PAR. Six weeks old plants were cold acclimated for one or three weeks (CA-SHORT and CA-LONG) in a growth chamber at 3°C, 12 h photoperiod and approximately 100 µmol m^−2 ^s^−1^ PAR from metal halide lamps. Six and nine weeks old non-acclimated plants (NA-SHORT and NA-LONG) or seven weeks old non-acclimated plants (NA) were included in experiment 1 and 2, respectively (see Table 1).

#### Inoculation and incubation

Sclerotia of *S. trifoliorum* were collected in the Norwegian EUCLEG field trial (located at Arneberg; 60°45’ N, 11°12’ E) in the spring of 2019. Individual sclerotia were surface sterilised, cut in slices, and allowed to produce mycelium on potato dextrose agar (PDA). After forming new sclerotia, the isolates were stored on PDA at a low positive temperature. Prior to each inoculation event, PDA plates were inoculated and placed at room temperature to initiate growth. Flasks with potato dextrose broth (PDB) were then inoculated with a few plugs with actively growing mycelium and kept at 9 °C. After one week, the medium was filtered away and the mycelium was homogenized in a 0.2% gelatine solution. Five isolates (named 202,887–202,891 and stored at the isolate collection of Norwegian Institute of Bioeconomy Research) were mixed in equal amounts and after dilution the resulting optical density at 430 nm was 0.5 in experiment 1, 3 and 4 and 0.15 in experiment 2. One ml was applied to the base of the petioles and surrounding soil. Non-inoculated controls (in experiment 1, 3 and 4) were mock inoculated with 1 ml of 0.2% gelatine. Plants were placed under an artificial snow cover consisting of layers of wet cellulose paper covered by a plastic sheet and incubated in darkness at 3 °C (all experiments) and 16 °C (experiment 2). Inoculated and non-inoculated plants were placed under separate covers. Artificial snow covers were removed prior to subsequent freezing treatment or regrowth.

#### Freezing treatments

Plants were exposed to freezing stress either before inoculation and incubation, or after. For this purpose, they were placed in programmed freezing chambers initially set at 2 °C. The temperature was first lowered from 2 °C to -3 °C at 1 °C h^−1^ and kept at this level for 12 h to ensure even freezing, after which the temperature was lowered again by 1 °C h^−1^ down to the assigned test temperature (-4.5 and − 6 °C in experiment 3 and − 4.5, -6 and − 7.5 °C in experiment 4). When the temperature reached the test temperature, it was kept there for 1 h before the temperature was raised, again by 1 °C h^−1^, up to 2 °C.

#### Recovery and measurements of survival and regrowth

After the designated incubation time the artificial snow covers were removed and plants were placed in a greenhouse (experiment 1 and 3) or a growth chamber (experiment 2 and 4) with the same conditions as during the pre-growth and allowed to recover and regrow. After four weeks, survival was recorded, and above-ground biomass was collected from surviving plants. Biomass from plants of the same population within each block was bulked, dried at 60 °C, and weighed. Average dry weight per tested plant (including dead ones) were calculated. In addition to the survival rate and dry matter of the regrowth, the relative regrowth was calculated for inoculated plants in experiment 1 as the dry matter divided by the dry matter of the non-inoculated plants of the same population receiving the same incubation and freezing treatments and averaged over replicates.

#### Statistical analysis of survival and regrowth

The experiments had a split-plot design, with inoculation and incubation length applied to main plots (i.e., tables on which all the plants were covered by an artificial snow cover), and freezing treatment applied to sub-plots. Within each sub-plot, populations were organized in randomly placed rows with five, eight, three or six plants per population (experiment 1–4, respectively). Survival rate, dry matter of the regrowth and relative regrowth were subjected to analyses of variance using PROC MIXED in SAS Enterprise Guide 7.1 (SAS Institute Inc., Cary, NC, USA). The statistical models are presented in Supplementary Tables 3, 4, 5, 6 and 7. The response variable values of the different treatment combinations were calculated as Least Square Means and contrasts among them were estimated using the Tukey-Kramer test implemented in PROC MIXED. Interaction effects between clover rot and freezing stress were studied further by testing the difference between the reduction in survival rate or regrowth in treatments involving both stresses and the reduction expected from additive effects only using PROC TTEST.

#### Analysis and comparison with results from other studies of the EUCLEG red clover collection

Larger sets of the EUCLEG red clover collection have been phenotyped in several field experiments [27] and in experiments under controlled conditions, including phenotyping of clover rot resistance of non-cold acclimated plants [24] and freezing tolerance of cold acclimated plants [28]. In the Norwegian field experiment, including 110 of the EUCLEG accessions [23], shoot growth in the establishment year was recorded with a plate meter at five points per plot in late September and in late October, in 2018. The following spring the field was naturally heavily infested with *S. trifoliorum* and the disease in each plot was recorded on a scale from 1 (all plants dead) to 9 (no symptoms).

Trait variables for which there was a significant effect of population in experiment 1 and 2 were included in correlation and principal component analysis (PCA), including data on shoot growth in the establishment year (two time points as well as the difference between them, which represents late autumn growth) and clover rot resistance as measured in the Norwegian field experiment, as well as freezing tolerance data from Zanotto et al. [28] and clover rot resistance data from Frey et al. [24]. Pearson correlation coefficients were calculated with the CORR procedure in SAS and PCA was performed in MiniTab v21.3.

### Supplementary Information

Supplementary Material 1.

Supplementary Material 2.

## Data Availability

The datasets used and/or analysed during the current study are available from the corresponding author on reasonable request.
