# Supplementary material for: Winter survival in red clover: experimental evidence for interactions among stresses
Source: BMC Plant Biol. 2024 May 28;24:467. doi: 10.1186/s12870-024-05167-5 (PMC11131274; doi:10.1186/s12870-024-05167-5)
Supplement: Supplementary file 1 — Supplementary Material 1. [file 12870_2024_5167_MOESM1_ESM.docx]

**Supplementary Fig. 1.** Overview of timeline in different treatments in experiment 1 (A) and 3 (B). Green, pregrowth or regrowth; blue, cold acclimation; pink, incubation under artificial snow cover; red, inoculation with *Sclerotinia trifoliorum*; blue circle; freezing event. NA-OLD, NA-YOUNG, CA-LONG and CA-SHORT; see Table 1 for an explanation. Experiment 2 was similar to experiment 1, except that there was only one non-acclimated (NA) treatment and instead two different incubation temperatures. Experiment 4 was similar to experiment 3, except that there were three different incubation lengths instead of two.
