# Supplementary material for: Winter survival in red clover: experimental evidence for interactions among stresses
Source: BMC Plant Biol. 2024 May 28;24:467. doi: 10.1186/s12870-024-05167-5 (PMC11131274; doi:10.1186/s12870-024-05167-5)
Supplement: Supplementary file 2 — Supplementary Material 2. [file 12870_2024_5167_MOESM2_ESM.docx]

**Supplementary Table 1.** Further details on the experiments described in Table 1.

|  | **Experiment 1** | **Experiment 2** | **Experiment 3** | **Experiment 4** |
| --- | --- | --- | --- | --- |
| Number of populations (see Table 2) | 9 | 12 | 2 | 2 |
| Number of repetitions of the whole experiment | 1 | 1 | 2 | 1 |
| Number of replicate “snow covers” per inoculation and incubation treatment | 3 | - | 3 | 2 (short incubation)  4 (long incubation) |
| Number of replicate sub-blocks per growth (exp. 1) or freezing treatment (exp. 3 and 4) within “snow covers” | 1 | - | 1 | 2 (3 for non-inoculated, non-frozen treatments) |
| Number of replicate “snow covers” per growth, inoculation and incubation treatment | - | 2 | - | - |
| Number of plants per population in each “snow cover” (exp. 2) or sub-block (exp. 1, 3 and 4) | 5 | 8 | 3 | 6 |

**Supplementary Table 2.** Populations tested

| **Population^1^** | **Name** | **Institution** | **Experiment** |
| --- | --- | --- | --- |
| EUC_TP_007 | S592 AberChianti | IBERS, UK | 1 |
| EUC_TP_008 | Gandalf | Graminor, Norway | 1, 3, 4 |
| EUC_TP_016 | Sangria | RAGT2n, France | 1 |
| EUC_TP_017 | Saija | Boreal, Finland | 1, 3 |
| EUC_TP_031 | NGB2487 | NordGen (origin: Sweden) | 1 |
| EUC_TP_034 | NGB4089 | NordGen (origin: Sweden) | 1 |
| EUC_TP_190 | Karim | RAGT2n, France | 1 |
| EUC_TP_231 | Trubadur | Agricultural Research, Ltd., Czech Republic | 1 |
| EUC_TP_234 | Vltavín | Agricultural Research, Ltd., Czech Republic | 1 |
| EUC_TP_002 | Discovery | INRA/AgriObtentions, France | 2 |
| EUC_TP_057 | Lanzenhaeusern_291 | Agroscope, Switzerland | 2 |
| EUC_TP_062 | Niederwangen_262 | Agroscope, Switzerland | 2 |
| EUC_TP_124 | GnRk0729 | Graminor AS, Norway | 2 |
| EUC_TP_125 | GnRk0747 | Graminor AS, Norway | 2 |
| EUC_TP_136 | LøRk9627 | Graminor AS, Norway | 2 |
| EUC_TP_153 | SW RK1119 | Lantmännen lantbruk, Sweden | 2 |
| EUC_TP_154 | SW RK1120 | Lantmännen lantbruk, Sweden | 2 |
| EUC_TP_165 | SWÅ RK09093 (Åke) | Lantmännen lantbruk, Sweden | 2 |
| EUC_TP_169 | Callisto | DLF Seed, Denmark | 2 |
| EUC_TP_184 | TPD-05-15-3127 | DLF Seed, Denmark | 2 |
| EUC_TP_189 | TPD-05-16-3177 | DLF Seed, Denmark | 2 |
| EUC_TP_004 | Milvus | Agroscope, Switzerland | 4 |

^1^ Population identity in the EUCLEG project

**Supplementary Table 3.** Analysis of variance of data obtained in experiment 1. A, survival rate of inoculated plants; B, dry weights of non-inoculated plants; C, dry weights of inoculated plants; D, relative dry weight (inoculated vs. non-inoculated). There were three replicate “snow covers” per incubation and inoculation treatment. Growth treatments were organized as four sub-blocks per “snow cover”, each sub-block containing five plants per population. Snow cover replicate x Incubation length and Snow cover replicate x Incubation length x Growth treatment were used as random factors in the model.

A

| **Effect** | **d.f.** | **Error d.f.** | **F-value** | **P-value** |
| --- | --- | --- | --- | --- |
| Incubation length | 1 | 4 | 21.4 | 0.01 |
| Growth treatment | 3 | 12 | 1.5 | 0.3 |
| Population | 8 | 140 | 3.0 | 0.004 |
| Incubation length x Growth treatment | 3 | 12 | 0.8 | 0.5 |
| Incubation length x Population | 8 | 140 | 0.9 | 0.5 |
| Growth treatment x Population | 24 | 140 | 1.2 | 0.2 |

^1^ degrees of freedom

B

| **Effect** | **d.f.** | **Error d.f.** | **F-value** | **P-value** |
| --- | --- | --- | --- | --- |
| Incubation length | 1 | 4 | 6.2 | 0.07 |
| Growth treatment | 3 | 12 | 40.7 | <0.0001 |
| Population | 8 | 143 | 1.9 | 0.07 |
| Incubation length x Growth treatment | 3 | 12 | 1.2 | 0.3 |
| Incubation length x Population | 8 | 143 | 1.4 | 0.2 |
| Growth treatment x Population | 24 | 143 | 1.8 | 0.02 |

C

| **Effect** | **d.f.** | **Error d.f.** | **F-value** | **P-value** |
| --- | --- | --- | --- | --- |
| Incubation length | 1 | 4 | 17.1 | 0.01 |
| Growth treatment | 3 | 12 | 1.6 | 0.2 |
| Population | 8 | 140 | 1.6 | 0.1 |
| Incubation length x Growth treatment | 3 | 12 | 1.2 | 0.4 |
| Incubation length x Population | 8 | 140 | 0.6 | 0.7 |
| Growth treatment x Population | 24 | 140 | 1.0 | 0.4 |

D

| **Effect** | **d.f.** | **Error d.f.** | **F-value** | **P-value** |
| --- | --- | --- | --- | --- |
| Incubation length | 1 | 4 | 15.7 | 0.02 |
| Growth treatment | 3 | 12 | 0.4 | 0.8 |
| Population | 8 | 140 | 2.2 | 0.03 |
| Incubation length x Growth treatment | 3 | 12 | 0.6 | 0.6 |
| Incubation length x Population | 8 | 140 | 0.8 | 0.6 |
| Growth treatment x Population | 24 | 140 | 1.1 | 0.4 |

**Supplementary Table 4.** Analysis of variance of survival rate in experiment 2. A) Incubation at 16 °C. B) Incubation at 3 °C. There were two replicate “snow covers” per growth treatment and incubation length, each containing 8 plants per population. Snow cover replicate x Incubation length x Growth treatment was used as a random factor in the model.

A

| **Effect** | **d.f.** | **Error d.f.** | **F-value** | **P-value** |
| --- | --- | --- | --- | --- |
| Incubation length | 2 | 9 | 99.3 | <0.0001 |
| Growth treatment | 2 | 9 | 37.8 | <0.0001 |
| Population | 11 | 143 | 2.7 | 0.003 |
| Incubation length x Growth treatment | 4 | 9 | 13.7 | 0.0007 |
| Incubation length x Population | 22 | 143 | 1.4 | 0.1 |
| Growth treatment x Population | 22 | 143 | 1.9 | 0.01 |

B

| **Effect** | **d.f.** | **Error d.f.** | **F-value** | **P-value** |
| --- | --- | --- | --- | --- |
| Incubation length | 2 | 9 | 16.0 | 0.001 |
| Growth treatment | 2 | 9 | 28.8 | 0.0001 |
| Population | 11 | 143 | 0.7 | 0.7 |
| Incubation length x Growth treatment | 4 | 9 | 1.5 | 0.3 |
| Incubation length x Population | 22 | 143 | 1.1 | 0.3 |
| Growth treatment x Population | 22 | 143 | 1.0 | 0.5 |

**Supplementary Table 5.** Analysis of variance of survival rate (A, B) and dry matter of regrowth (C, D) in experiment 3. Two replicate experiments were conducted. In each of them, plants were organized in a split-plot design, with inoculation and incubation length applied to three replicate “snow covers” per combination, and freezing treatment before or after inoculation applied to sub-blocks within snow covers. There were three plants for each of the two populations in each sub-block. As regrowth occurred in a greenhouse at different timepoints during the season, with a confounding effect on the factor incubation length, analyses were performed separately for the short (A, C) and long (B, D) incubation. Replicate experiment, and Replicate experiment x Replicate snow cover x Inoculation treatment were used as random factors in the model.

A

| **Effect** | **d.f.** | **Error d.f.** | **F-value** | **P-value** |
| --- | --- | --- | --- | --- |
| Inoculation treatment | 1 | 9 | 2.2 | 0.2 |
| Freezing treatment | 4 | 94 | 2.1 | 0.09 |
| Population | 1 | 94 | 0.1 | 0.8 |
| Inoculation treatment x Freezing treatment | 4 | 94 | 2.9 | 0.03 |
| Inoculation treatment x Population | 1 | 9 | 0.1 | 0.8 |
| Freezing treatment x Population | 4 | 94 | 0.3 | 0.9 |

B

| **Effect** | **d.f.** | **Error d.f.** | **F-value** | **P-value** |
| --- | --- | --- | --- | --- |
| Inoculation treatment | 1 | 9 | 84.0 | <0.0001 |
| Freezing treatment | 4 | 94 | 5.4 | 0.0006 |
| Population | 1 | 94 | 2.7 | 0.1 |
| Inoculation treatment x Freezing treatment | 4 | 94 | 3.0 | 0.02 |
| Inoculation treatment x Population | 1 | 9 | 1.9 | 0.2 |
| Freezing treatment x Population | 4 | 94 | 0.5 | 0.8 |

C

| **Effect** | **d.f.** | **Error d.f.** | **F-value** | **P-value** |
| --- | --- | --- | --- | --- |
| Inoculation treatment | 1 | 9 | 2.5 | 0.1 |
| Freezing treatment | 4 | 94 | 20.2 | <0.0001 |
| Population | 1 | 94 | 1.5 | 0.2 |
| Inoculation treatment x Freezing treatment | 4 | 94 | 0.9 | 0.5 |
| Inoculation treatment x Population | 1 | 9 | 1.3 | 0.3 |
| Freezing treatment x Population | 4 | 94 | 1.8 | 0.1 |

D

| **Effect** | **d.f.** | **Error d.f.** | **F-value** | **P-value** |
| --- | --- | --- | --- | --- |
| Inoculation treatment | 1 | 9 | 53.3 | <0.0001 |
| Freezing treatment | 4 | 94 | 15.1 | <0.0001 |
| Population | 1 | 94 | 0.1 | 0.8 |
| Inoculation treatment x Freezing treatment | 4 | 94 | 0.7 | 0.6 |
| Inoculation treatment x Population | 1 | 9 | 0.2 | 0.6 |
| Freezing treatment x Population | 4 | 94 | 0.1 | 1.0 |

**Supplementary Table 6.** Analysis of variance of survival rate (A) and dry matter of regrowth (B) in experiment 4. Plants were organized in a split-plot design, with inoculation and incubation length applied to two (short incubation) or four (long incubation) replicate “snow covers” per combination. Within each snow cover, freezing treatments were applied to sub-blocks (two per freezing treatment). There were six plants for each of the two populations in each sub-block. Replicate snow cover x Incubation length x Inoculation treatment was used as a random factor in the model.

A

| **Effect** | **d.f.** | **Error d.f.** | **F-value** | **P-value** |
| --- | --- | --- | --- | --- |
| Incubation | 1 | 8 | 1.4 | 0.3 |
| Inoculation | 1 | 8 | 49.0 | 0.0001 |
| Freezing | 6 | 302 | 74.2 | <0.0001 |
| Population | 1 | 302 | 1.9 | 0.2 |
| Incubation x Inoculation | 1 | 8 | 1.4 | 0.3 |
| Incubation x Freezing | 6 | 302 | 2.6 | 0.02 |
| Incubation x Population | 1 | 302 | 0.4 | 0.5 |
| Inoculation x Freezing | 6 | 302 | 15.3 | <0.0001 |
| Inoculation x Population | 1 | 302 | 0.2 | 0.7 |
| Freezing x Population | 6 | 321 | 1.7 | 0.1 |
| Incubation x Inoculation x Freezing | 6 | 302 | 4.0 | 0.0007 |
| Incubation x Inoculation x Population | 1 | 302 | 0.0 | 0.9 |
| Incubation x Freezing x Population | 6 | 302 | 1.6 | 0.1 |
| Inoculation x Freezing z Population | 1 | 302 | 0.8 | 0.6 |

B

| **Effect** | **d.f.** | **Error d.f.** | **F-value** | **P-value** |
| --- | --- | --- | --- | --- |
| Incubation | 1 | 8 | 0.3 | 0.6 |
| Inoculation | 1 | 8 | 32.5 | 0.0005 |
| Freezing | 6 | 302 | 177.9 | <0.0001 |
| Population | 1 | 302 | 0.6 | 0.5 |
| Incubation x Inoculation | 1 | 8 | 0.8 | 0.4 |
| Incubation x Freezing | 6 | 302 | 54.4 | <0.0001 |
| Incubation x Population | 1 | 302 | 9.8 | 0.002 |
| Inoculation x Freezing | 6 | 302 | 2.3 | 0.03 |
| Inoculation x Population | 1 | 302 | 0.3 | 0.6 |
| Freezing x Population | 6 | 321 | 0.8 | 0.6 |
| Incubation x Inoculation x Freezing | 6 | 302 | 2.3 | 0.03 |
| Incubation x Inoculation x Population | 1 | 302 | 0.1 | 0.7 |
| Incubation x Freezing x Population | 6 | 302 | 1.1 | 0.4 |
| Inoculation x Freezing x Population | 1 | 302 | 0.5 | 0.8 |

**Supplementary Table 7.** Analysis of variance of survival rate (A) and dry matter of regrowth (B) in experiment 4 (see Supplementary Table 6), excluding the non-frozen controls and splitting the freezing treatment into two factors; the freezing temperature and the timing of freezing (before and after incubation).

A

| **Effect** | **d.f.** | **Error d.f.** | **F-value** | **P-value** |
| --- | --- | --- | --- | --- |
| Incubation | 1 | 8 | 1.1 | 0.3 |
| Inoculation | 1 | 8 | 56.6 | <0.0001 |
| Freezing temperature | 2 | 243 | 34.2 | <0.0001 |
| Timing of freezing | 1 | 243 | 211.4 | <0.0001 |
| Population | 1 | 243 | 1.9 | 0.2 |
| Incubation x Inoculation | 1 | 8 | 1.2 | 0.3 |
| Incubation x Freezing temperature | 2 | 243 | 0.7 | 0.5 |
| Incubation x Timing of freezing | 1 | 243 | 10.7 | 0.001 |
| Incubation x Population | 1 | 243 | 0.5 | 0.5 |
| Inoculation x Freezing temperature | 2 | 243 | 0.7 | 0.5 |
| Inoculation x Timing of freezing | 1 | 243 | 59.2 | <0.0001 |
| Inoculation x Population | 1 | 243 | 0.1 | 0.8 |
| Freezing temperature x Timing of freezing | 2 | 243 | 13.6 | <0.0001 |
| Freezing temperature x Population | 2 | 243 | 1.6 | 1.2 |
| Timing of freezing x Population | 1 | 243 | 3.9 | 0.05 |
| Incubation x Inoculation x Freezing temperature | 2 | 243 | 3.8 | 0.02 |
| Incubation x Inoculation x Timing of freezing | 1 | 243 | 0.1 | 0.7 |
| Incubation x Inoculation x Population | 1 | 243 | 0.0 | 1.0 |
| Incubation x Freezing temperature x Timing of freezing | 2 | 243 | 0.4 | 0.7 |
| Incubation x Freezing temperature x Population | 2 | 243 | 2.4 | 0.1 |
| Incubation x Timing of freezing x Population | 1 | 243 | 3.6 | 0.06 |
| Inoculation x Freezing temperature x Timing of freezing | 2 | 243 | 2.1 | 0.1 |
| Inoculation x Freezing temperature x Population | 2 | 243 | 0.6 | 0.5 |
| Inoculation x Timing of freezing x Population | 1 | 243 | 1.2 | 0.3 |
| Freezing temperature x Timing of freezing x Population | 2 | 243 | 0.5 | 0.6 |

B

| **Effect** | **d.f.** | **Error d.f.** | **F-value** | **P-value** |
| --- | --- | --- | --- | --- |
| Incubation | 1 | 8 | 8.1 | 0.02 |
| Inoculation | 1 | 8 | 27.5 | 0.0008 |
| Freezing temperature | 2 | 243 | 25.3 | <0.0001 |
| Timing of freezing | 1 | 243 | 353.6 | <0.0001 |
| Population | 1 | 243 | 2.1 | 0.2 |
| Incubation x Inoculation | 1 | 8 | 1.0 | 0.3 |
| Incubation x Freezing temperature | 2 | 243 | 11.5 | <0.0001 |
| Incubation x Timing of freezing | 1 | 243 | 123.3 | <0.0001 |
| Incubation x Population | 1 | 243 | 10.5 | 0.001 |
| Inoculation x Freezing temperature | 2 | 243 | 1.7 | 0.2 |
| Inoculation x Timing of freezing | 1 | 243 | 0.0 | 0.9 |
| Inoculation x Population | 1 | 243 | 0.1 | 0.7 |
| Freezing temperature x Timing of freezing | 2 | 243 | 3.0 | 0.05 |
| Freezing temperature x Population | 2 | 243 | 0.6 | 0.6 |
| Timing of freezing x Population | 1 | 243 | 0.0 | 0.9 |
| Incubation x Inoculation x Freezing temperature | 2 | 243 | 0.2 | 0.8 |
| Incubation x Inoculation x Timing of freezing | 1 | 243 | 10.0 | 0.002 |
| Incubation x Inoculation x Population | 1 | 243 | 0.0 | 0.8 |
| Incubation x Freezing temperature x Timing of freezing | 2 | 243 | 2.3 | 0.1 |
| Incubation x Freezing temperature x Population | 2 | 243 | 0.6 | 0.6 |
| Incubation x Timing of freezing x Population | 1 | 243 | 5.7 | 0.02 |
| Inoculation x Freezing temperature x Timing of freezing | 2 | 243 | 2.2 | 0.1 |
| Inoculation x Freezing temperature x Population | 2 | 243 | 0.4 | 0.7 |
| Inoculation x Timing of freezing x Population | 1 | 243 | 3.3 | 0.07 |
| Freezing temperature x Timing of freezing x Population | 2 | 243 | 0.0 | 1.0 |

**Supplementary Table 8.** Pearson correlation coefficients among traits recorded in the set of 110 accessions of the EUCLEG red clover panel (A), the traits recorded in the set of nine accessions used in experiment 1 (B) and experiment 2 (C).

A

|  | Canopy height late October | Change in canopy height | Clover rot resistance, field^4^ | Clover rot resistance, controlled conditions^1^ | Freezing tolerance^2^ |
| --- | --- | --- | --- | --- | --- |
| Canopy height late September | **0.84**  **(<0.0001)** | **0.52**  **(<0.0001)** | **-0.56**  **(<0.0001)** | **-0.37**  **(<0.0001)** | **-0.29 (0.002)** |
| Canopy height late October |  | **0.89**  **(<0.0001)** | **-0.59**  **(<0.0001)** | **-0.50**  **(<0.0001)** | **-0.34**  **(0.0003)** |
| Change in canopy height |  |  | **-0.47**  **(<0.0001)** | **-0.49**  **(<0.0001)** | **-0.31**  **(0.001)** |
| Clover rot resistance, field |  |  |  | **0.34**  **(0.0003)** | **0.19**  **(0.04)** |
| Clover rot resistance, controlled conditions^1^ |  |  |  |  | 0.17  (0.08) |

^1^ Non-acclimated plants; data from Frey et al. (2022); ^2^ Measured as -LT50; data from Zanotto et al. (2021b, 2023)

B

|  | Canopy height late October^1^ | Change in canopy height | Clover rot resistance, field^1^ | Clover rot resistance, controlled conditions^2^ | Survival rate of inoculated plants^3^ | Regrowth of non-inoculated NA-OLD^4^ | Freezing tolerance^1^ |
| --- | --- | --- | --- | --- | --- | --- | --- |
| Canopy height late September | **0.86 (0.003)** | 0.35 (0.4) | -0.44  (0.2) | **-0.68 (0.04)** | -0.46 (0.2) | 0.42 (0.3) | -0.05 (0.9) |
| Canopy height late October^1^ |  | **0.78 (0.01)** | -0.41  (0.3) | **-0.67**  **(0.05)** | -0.51  (0.2) | **0.68**  **(0.04)** | -0.02 (1.0) |
| Change in canopy height |  |  | -0.22 (0.6) | -0.40 (0.3) | -0.37 (0.3) | **0.74 (0.02)** | 0.03 (0.9) |
| Clover rot resistance, field^1^ |  |  |  | **0.71**  **(0.03)** | 0.58  (0.1) | **-0.77**  **(0.01)** | 0.04 (0.9) |
| Clover rot resistance, controlled conditions^2^ |  |  |  |  | **0.76**  **(0.02)** | -0.59  (0.1) | -0.11 (0.8) |
| Survival rate of inoculated plants^3^ |  |  |  |  |  | -0.60  (0.09) | -0.55 (0.1) |
| Regrowth of non-inoculated NA-OLD^4^ |  |  |  |  |  |  | -0.05 (0.9) |

^1^ Measured as -LT50; data from Zanotto et al. (2021b, 2023); ^2^ Non-acclimated plants; data from Frey et al. (2022); ^3^ Across growth treatments and incubation lengths; ^4^ Across incubation lengths

C

|  | Canopy height late October^1^ | Change in canopy height | Clover rot resistance, field^1^ | Clover rot resistance, controlled conditions^2^ | Survival rate of inoculated plants^3^ | Freezing tolerance^1^ |
| --- | --- | --- | --- | --- | --- | --- |
| Canopy height late September | **0.84 (0.0005)** | **0.58**  **(0.05)** | **-0.72**  **(0.008)** | -0.26 (0.4) | -0.35 (0.3) | -0.17 (0.6) |
| Canopy height late October^1^ |  | **0.92 (<0.0001)** | **-0.84**  **(0.0005)** | -0.35  (0.3) | -0.34  (0.3) | -0.35 (0.3) |
| Change in canopy height |  |  | **-0.78 (0.003)** | -0.34 (0.3) | -0.27 (0.4) | -0.42 (0.2) |
| Clover rot resistance, field^1^ |  |  |  | 0.23  (0.5) | 0.28  (0.4) | 0.47 (0.1) |
| Clover rot resistance, controlled conditions^2^ |  |  |  |  | **0.61**  **(0.04)** | 0.11 (0.7) |
| Survival rate of inoculated plants^3^ |  |  |  |  |  | -0.06 (0.8) |

^1^ Measured as -LT50; data from Zanotto et al. (2021b, 2023); ^2^ Non-acclimated plants; data from Frey et al. (2022); ^3^ Across growth treatments, incubation temperatures and lengths
